# Scoping the psychological support practices of Australian health professionals working with people with primary brain tumor and their families

**DOI:** 10.1002/pon.5929

**Published:** 2022-04-02

**Authors:** Tamara Ownsworth, Katarzyna Lion, Ursula M. Sansom‐Daly, Kerryn Pike, Eng‐Siew Koh, Georgia K. B. Halkett, Mark B. Pinkham, Raymond J. Chan, Haryana M. Dhillon, for the BRAINS Program Investigators

**Affiliations:** ^1^ School of Applied Psychology & The Hopkins Centre Menzies Health Institute Queensland Griffith University Brisbane Queensland Australia; ^2^ School of Women's and Children's Health UNSW Medicine and Health UNSW Sydney Kensington New South Wales Australia; ^3^ Behavioural Sciences Unit Kids Cancer Centre Sydney Children's Hospital Randwick New South Wales Australia; ^4^ Sydney Youth Cancer Service Nelune Comprehensive Cancer Centre Prince of Wales Hospital Randwick New South Wales Australia; ^5^ School of Psychology & Public Health & John Richards Centre for Rural Ageing Research La Trobe University Victoria Australia; ^6^ South Western Sydney Clinical School University of New South Wales Faculty of Medicine Sydney New South Wales Australia; ^7^ Liverpool and Macarthur Cancer Therapy Centres Liverpool New South Wales Australia; ^8^ Ingham Institute for Applied Medical Research Liverpool New South Wales Australia; ^9^ Curtin School of Nursing Faculty of Health Sciences Curtin University Perth Western Australia Australia; ^10^ School of Medicine University of Queensland Woolloongabba Queensland Australia; ^11^ Department of Radiation Oncology Princess Alexandra Hospital Woolloongabba Queensland Australia; ^12^ Caring Futures Institute College of Nursing and Health Sciences Flinders University Bedford Park South Australia Australia; ^13^ School of Psychology, Faculty of Science Psycho‐Oncology Cooperative Research Group University of Sydney Sydney New South Wales Australia; ^14^ School of Psychology, Faculty of Science Centre for Medical Psychology & Evidence‐based Decision‐making University of Sydney Sydney New South Wales Australia

**Keywords:** brain tumor, cancer, health professionals, psycho‐oncology, psychosocial support

## Abstract

**Objectives:**

This study aimed to scope the psychological support practices of Australian health professionals providing supportive care to adults with primary brain tumor.

**Method:**

Health professionals from multidisciplinary organizations and cancer support services completed an online survey focused on psychological support for people with brain tumor (PwBT) and family members, and perceived barriers or gaps in support provision.

**Results:**

107 professionals, mainly from psychology (45%), nursing (20%), and social work (10%) backgrounds, completed the survey. Scope of practice differed according to discipline, with psychologists and nurses most likely to screen for psychological distress (71%–76%), and psychologists more typically providing at least one psychological support session (78%). Psychologists were more likely to screen for cognitive impairment (31%), whereas nurses and social workers more commonly provided family‐based support (62%–73%). Psychological support was more frequently provided in the long‐term management phase (78%) than early post‐diagnosis/treatment (45%). System‐level barriers to accessing psychological support were most frequently identified, which included limited resources and funding, insufficient staff time, lengthy waitlists and costs, poor service coordination, and lack of staff with brain tumor‐specific training.

**Conclusions:**

The provision of psychological support for PwBT varies according to discipline, setting and management phase. Further research on different models of psychosocial care is needed to inform strategies to address organizational and policy factors impacting professionals' scope of practice.

## 1 INTRODUCTION

Primary brain cancer is a rare (3.5/100,000),^1^ yet serious illness. The diverse physical, cognitive, emotional, and behavioral effects related to the tumor and/or treatment can affect people's psychological well‐being, independence, and quality of life.^2^ Prognosis is generally poor with five‐year survival around 36% for high‐grade glioma.^3^ Further, 80% of lower‐grade gliomas recur and/or progress to malignant disease within 10 years.^4^ Despite a better prognosis, individuals with benign tumor can experience serious functional deficits, and face challenges associated with long‐term survivorship.^2^ High rates of psychological distress are evident across the illness trajectory,^5^, ^6^, ^7^, ^8^ as people with brain tumor (PwBT) and family members face ongoing stressors related to the uncertain prognosis, fear of recurrence, and functional decline.

Clinical practice guidelines for cancer^9^, ^10^, ^11^ and brain tumor^12^, ^13^ emphasize the need for routine screening for distress and unmet needs, followed by tailored psychosocial care to address individual and family members' information, emotional, existential, spiritual, social, and practical support needs from early post‐diagnosis to long‐term survivorship or end‐of‐life. Such support may relate to understanding of and coping with the diagnosis, threat to life, and functional consequences.^2^ Family members frequently report emotional and behavioral changes in PwBT and have their own distinct support needs related to caregiving responsibilities and uncertainty about the future.^14^, ^15^, ^16^

Psychological support following brain tumor encompasses assessment of cognitive, behavioral, and emotional functioning, education, individual and family counseling, psychotherapy, and rehabilitation.^12^, ^17^ Flexible and timely access to psychological support is essential given individuals' readiness and preferences for support change across the course of the illness.^18^ Moreover, care pathways need to address psychological support needs from diagnosis through to long‐term survivorship and/or end‐of‐life care.^9^, ^12^

Evidence for interventions improving psychological adjustment and quality of life for PwBT is growing, with research demonstrating the efficacy of psychotherapy and rehabilitation delivered in the home,^19^ clinic settings,^20^ and via telehealth.^17^ Uptake of telehealth interventions has been higher when involving real‐time interaction with health professionals compared with self‐guided online interventions.^17^ There is also preliminary support for the efficacy of psychological interventions involving both PwBT and caregivers^21^, ^22^, ^23^ and those specifically targeting caregivers' information and support needs.^24^

Assessment and rehabilitation of cognitive impairments is another integral aspect of psychological management after brain tumor. Although most marked during active treatment, cognitive deficits can persist longer‐term or increase in the context of functional decline.^25^ Neuropsychological assessment provides an objective indication of individuals' functional status and informs tailored rehabilitation.^26^ Cognitive assessment guidelines have been developed for brain tumor,^27^ with a specific battery recommended by the International Cognition and Cancer Task Force (ICCTF)^28^ to monitor cancer‐related changes in cognition. Several studies have found cognitive rehabilitation to be effective for improving cognitive functioning^20^, ^29^; however, it is unknown how frequently such interventions are implemented in practice.

Multidisciplinary approaches to psychological support are strongly advocated, with recognition that medical, nursing, and allied health professionals can have differing yet complementary roles in provision of supportive care.^2^, ^9^ A recent systematic review found that care coordination and continuity, personalized information, and effective communication increased individuals' sense of support at diagnosis, irrespective of discipline.^30^ Little is known, however, about the scope of practice of professionals involved in psychological support and whether this differs according to discipline and illness phase. Further, despite established guidelines for psychosocial care in cancer and brain tumor^11^, ^12^ and evidence supporting the efficacy of interventions,^17^ various barriers or gaps may exist for implementation in practice.^13^

### 1.1 Study aims

This study was designed to scope the psychological support practices of Australian health professionals working with adults with primary brain tumor to deliver supportive care. We aimed to identify the frequency and nature of four recommended practices: distress screening, cognitive assessment, provision of psychological support to PwBT, and psychological support to family members. A further aim was to identify barriers to access or gaps in the provision of psychological support for the brain tumor population.

## 2 METHODS

### 2.1 Study design

The study involved a cross‐sectional survey comprising closed and open‐ended response options, with participants able to volunteer for a subsequent semi‐structured interview (reported elsewhere). We aimed to recruit a minimum of 100 health professionals representing various disciplines across different states in Australia.

### 2.2 Participants & procedure

The study received ethical approval from the Griffith University Human Research Ethics Committee (GUHREC Ref No: 2021/163). Australian health professionals who provide supportive care to PwBT (defined as approaches, interventions and services aiming to improve care and QoL by meeting individual and family members' information, emotional, spiritual, social or practical support needs across the disease trajectory^17^) regardless of frequency, were eligible to participate. Professions included psychology, nursing, social work, oncology (e.g., medical, neuro‐oncology & radiation oncology), occupational therapy, speech pathology, and psychiatry. Participants were recruited from cancer support services, researchers' professional networks, and multidisciplinary organizations in psycho‐oncology, neuro‐oncology, brain injury, and palliative care. Organizations were asked to distribute the survey link to members via newsletters, websites, and/or email. The survey was piloted by psychology (*n* = 3) and nursing (*n* = 1) professionals with minor feedback on wording incorporated prior to final dissemination. The survey was administered between April and August 2021 via the Griffith University Online Research Survey Tool, powered by LimeSurvey (Version 2.59).

### 2.3 Materials

After information and consent, the survey consisted of 37 questions with a fixed‐choice format and ‘other’ option. The first section collected demographic data including age, gender, ethnicity, first language, state of residence, discipline, and highest qualification. The second section included questions on work setting, main clinical population/s, experience with brain tumor population (including benign, malignant, or both subtypes; illness phase), and frequency and nature of distress screening, cognitive assessment and psychological support interventions provided to PwBT and family (see Supplemental Material 1). Responses for illness phase were coded into three groups: (1) post‐diagnosis/acute treatment; (2) long‐term management (including long‐term survivorship and end‐of‐life care); and (3) across the illness trajectory (working from acute inpatient through to long‐term management). For frequency of practice, response options related to the proportion of all PwBT seen as clients: *never (0% of clients seen)*, *rarely (10%–20%)*, *sometimes (30%–50%)*, *almost always (60%–80%),* and *always (∼100%)*. A final open‐ended question inquired about perceived barriers or gaps in the provision of psychological support for PwBT and families. Participants were invited to enter a random prize draw for free registration at a cancer conference.

### 2.4 Data analysis

Data screening and analysis were conducted using the Statistical Package for the Social Sciences for Windows (v27.0). Descriptive statistics summarized the frequency and types of distress screening, cognitive assessment, and psychological support for PwBT and family members. The chi‐square test of independence or Fisher's Exact Probability Test (cell size <5) examined differences in practice according to discipline, work setting, and illness phase. For these analyses, responses were recoded as ‘infrequently’ (never; rarely) or ‘frequently’ (sometimes; almost always; always). Open‐ended question responses were coded into categories using content analysis by two authors (KL and TO), with the frequency of responses collated. Quotes were used in the results to illustrate responses for categories in which at least 10% of participants' comments were coded. All data are available upon request from the first author.

## 3 RESULTS

### 3.1 Sample characteristics

Of the 135 people who accessed the online survey, 107 health professionals were included in the analysis. Twenty‐eight were ineligible (not working with PwBT) or did not provide sufficient responses to core questions. As shown in Table 1, most were female (86%) and lived in Queensland (36%), Victoria (23%), or New South Wales (20%). The main disciplines represented were psychology (42%), nursing (20%), social work (10%), occupational therapy (8%), and oncology (8%). Most participants worked in a hospital setting (71%) and their highest qualification was a master's degree (40%). The highest proportion had 11–20 years of experience (42%) and worked with PwBT monthly or weekly (47%), providing care across all illness phases (52%). Approximately one third of professionals worked in oncology or cancer care settings (35%), whereas two thirds worked in settings other than oncology, including brain injury (14%), end‐of‐life (14%), chronic health (11%), mental health (10%), and neurosurgery (8%).

**TABLE 1:** Demographic and work characteristics (*n* = 107)

	Sample characteristics	N	%
Age (years)	26–35	16	15.0
	36–45	33	30.8
	46–55	30	28.0
	>55	28	26.2
Gender	Male	15	14.0
	Female	92	86.0
Ethnic background	Australian	89	83.2
	New Zealander	2	1.9
	Asian	5	4.7
	European	7	6.5
	African	2	1.9
	Other	2	1.9
First language spoken as a child	English	101	94.4
	Other	5	4.7
State	Queensland	38	35.5
	New South Wales	21	19.6
	Australian Capital Territory	1	0.9
	Victoria	25	23.4
	Tasmania	5	4.7
	South Australia	6	5.6
	Northern Territory	1	0.9
	Western Australia	9	8.4
	Missing	1	0.9
Qualification	Bachelor's degree +/− honors	34	31.8
	Masters	43	40.2
	Doctorate/PhD	25	23.4
	Other	4	3.7
	Missing	1	0.9
Discipline	Psychology	45	42.1
	General	3	2.8
	Clinical psychology	22	20.6
	Health psychology	2	1.9
	Neuropsychology	18	16.8
	Psychiatry	3	2.8
	Oncology	9	8.4
	Nursing	21	19.6
	Social work	11	10.3
	Occupational therapy	8	7.5
	Other	10	9.3
Client population	Adults only	81	75.7
	Adults diagnosed with brain tumor <18 years	5	4.7
	Adolescents and young adults	2	1.8
	Any ages	19	17.8
Clinical setting^a^	Private practice	23	15.3
	Hospital – Inpatient	39	26.0
	Hospital – Outpatient	68	45.3
	Government department	2	1.3
	Community organization	13	8.7
	University	2	1.3
	Other	3	2.0
Frequency of working with adults diagnosed with brain tumor	Once a year or less	5	4.7
	A few times a year	18	16.8
	Every couple of months	21	19.6
	At least once per month	21	19.6
	Weekly	29	27.1
	Daily	13	12.1
Years of experience with adults with brain tumor	<2	10	9.3
	2–10	40	37.4
	11–20	45	42.1
	>20	12	11.1
Brain tumor types	Benign or lower grade	6	5.6
Mainly worked with	Malignant or high grade	65	60.7
	Benign and malignant	22	20.6
	Metastatic tumor	8	7.5
	Other	6	5.6
Phase of illness	Early post‐diagnosis/treatment	23	22
	Long‐term post‐treatment	28	26
	Across all illness phases	56	52

^a^ Multiple responses possible.

### 3.2 Scope of practice

**FIGURE 1:**
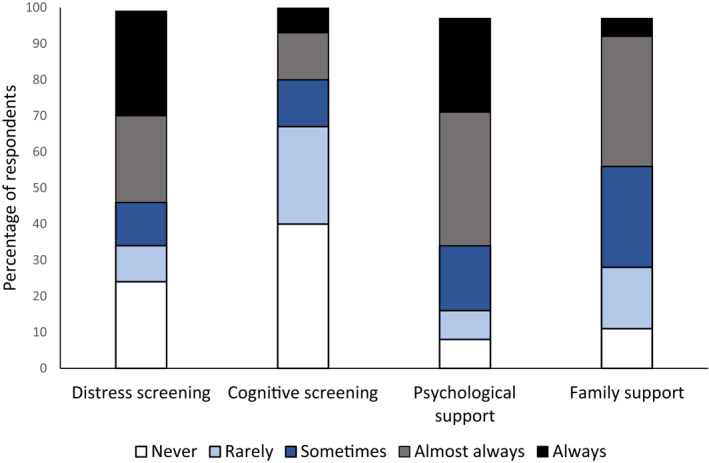
Frequency of psychological support practices (Note: Frequency was rated as a proportion of all clients seen with primary brain tumor)

***Psychological distress screening.*** About two thirds of professionals (65%) indicated they frequently screen for distress in PwBT or family members (see Figure 1). Instruments most used were the Distress Thermometer (41%), Depression Anxiety Stress Scales‐21 (25%), and Hospital Anxiety and Depression Scale (14%). Administration was usually paper‐based (59%) or via telephone (20%). Psychologists (71%, *p* = 0.013) and nurses (76%, *p* = 0.021) were more likely to screen for distress than social workers (27%). Professionals supporting PwBT in the longer‐term illness phase were more likely to screen for distress (79%) than those supporting PwBT across all illness phases (57%, *p* = 0.02; Supplementary Tables 1 and 2).

***Cognitive assessment.*** One third (33%) indicated frequently conducting formal cognitive assessment with PwBT. Verbal Fluency Test (18%), Trail Making Test (16%), Wechsler Adult Intelligence Scale‐IV (13%), Montreal Cognitive Assessment (13%), and Wechsler Memory Scale‐IV or subscales (13%) were most used. Psychologists (31%), and neuropsychologists specifically (78%), were more likely to assess cognitive function than social workers (0%, *p* = 0.049). Professionals working in oncology (14%) were less likely to conduct cognitive assessments than those working in other settings (45%, *p* = 0.004), such as brain injury and neurosurgery. Professionals working in the early post‐diagnosis/treatment phase (30%, *p* = 0.012) and those providing long‐term management (36%, *p* = 0.02) were more likely to assess cognitive function than those working with PwBT across the illness trajectory (7%; Supplementary Tables 3 and 4).

***Psychological support for PwBT.*** Eighty‐one percent of professionals frequently provided at least one psychological support session to PwBT. The highest proportion provided 2‐8 sessions (64%). Psychological support was more typically provided by psychologists (78%) than nurses (42%, *p* = 0.009), and by professionals working in the long‐term management phase (78%) and across the illness trajectory (70%) as compared to early post‐diagnosis/treatment (45%; *p* = 0.02; *p* = 0.041 respectively). Psychological support was most typically delivered in‐person (48%), followed by a mix of telehealth and face‐to‐face (28%). Psychoeducation (25%) and supportive counseling (23%) were the most common interventions. Cognitive rehabilitation was provided by 14%, which mainly involved individual compensatory training (72%). Of those delivering psychological therapy (11%), acceptance and commitment therapy (22%) and cognitive behavioral therapy (19%) were most common (Supplementary Tables 5, 7‐10).

***Psychological support for family members.*** Over two thirds (68%) indicated providing psychological support to family members. The highest proportion provided 1‐3 sessions (48%). Psychologists (56%) were less likely to provide psychological support to family members than nurses (90%, *p* = 0.01) or social workers (91%, *p* = 0.009). Professionals in oncology or cancer care settings (48%) were more likely to provide support to family members than those not working in oncology settings (16%, *p* = 0.019; Supplementary Table 6).

### 3.3 Perceived barriers and gaps

Seventy‐six participants identified barriers to PwBT or family members accessing psychological support. Of the 11 subcategories identified (see Supplementary Table 11), eight depicted *system‐level barriers or gaps,* which were identified by most participants (82%), and three were *barriers related to PwBT,* which were identified by 30% of participants.

***System‐level barriers:*** Limited resources and funding for services specific to PwBT and families (28%) was most frequently identified, followed by insufficient time or capacity within staffing (26%), lack of post‐treatment rehabilitation and support services (25%), lengthy waitlists and costs to the patient (24%), poor coordination or integration between services (22%), lack of staff with training or experience with brain tumor (21%), lack of awareness of services available or how to access these (13%), and limited support for family members (11%).

Limited resources and funding was typically related to psychology or psycho‐oncology services, including the lack of scope to refer to neuropsychologists and psychiatrists; *A dedicated psychology service for the neurological cancer service at the hospital would allow for better multidisciplinary team care/integrated care for patients* (P057); *Neuropsychological assessment to provide strategies to the patient & carer, especially for patients who live many years* (P081)*; There is a terrible shortage of psychiatrists to manage the complex acquired serious mental health issues that can occur* (P105). Poor coordination and integration between services was often attributed to the lack of neuro‐oncology care coordinators, needed to provide *ongoing supportive care and navigation of the health care pathway for the patient and family/carers post completion of treatment* (P007).

Lack of staff time and capacity affected professionals' ability to provide the level of support perceived as needed; *They have high needs, glioblastomas (GBMs) should probably be automatically referred (though we'd need more staff for this;* P043); *Insufficient time to provide feedback, cognitive strategies, and counseling to both patient and family* (P083). Further, delays in support and long waitlists and the costs of accessing specialized psychological support were common barriers; *No neuropsychology at (service) + long waitlist elsewhere* (P034); *Disability service) applications take too long and are not responsive to people with progressive and deteriorating conditions* (P069).

Other health professionals' lack of training and/or experience in working with PwBT was also perceived as a barrier to providing appropriate psychological support; *Cancer‐focused psychologists aren't always equipped to understand the cognitive effects of brain tumor* (P039). In other instances, professionals were not aware of services to refer PwBT to or how they could access these services; *Unclear referral processes and knowledge of support services available* (P072). Services available for people with other neurological conditions were not always perceived to meet the needs of PwBT or their families, and psychological support for family members was an identified gap; *Carer support programs specific to primary brain tumors are needed rather than dementia/brain injury* (P071); *Families would also benefit from more access to psychology* (P045).

***Barriers related to PwBT:*** These included tumor characteristics and prognosis impacting service access (13%), lack of acceptance of counseling and functional deficits (12%), and travel and transport issues (5%). Hospital‐based psychological support was sometimes unavailable for people with benign tumors; *There appears to be variation in what allied health services the person can access depending on whether the tumor is benign or malignant ‐ even those with non‐malignant brain tumors require psycho‐oncology management* (P014). Conversely, individuals with high‐grade or malignant tumors may not have access to brain injury rehabilitation services or longer‐term follow‐up in the community. *Acquired brain injury services (who might be best placed to provide psychological support) often don't accept people with brain cancer due to prognosis* (P039). *Limited supports for GBM patients who often leave hospital with minimal impairments but then decline rapidly in the community* (P013).

Some professionals perceived that individuals' lack of acceptance of the need for counseling or their functional deficits impeded their access to support and limited the types of interventions they could receive; *Patient acceptance of need/benefit, fatigue, communication issues induced by disease* (P071); *Significant cognitive and/or communication impairment limits the interventions that can be used and requires altering of the interventions that remain* (P103).

## 4 DISCUSSION

We identified that most Australian professionals working with PwBT (81%) provide at least one session of psychological support (most commonly psychoeducation or supportive counseling) to each individual. Support for family members (68%) and distress screening (65%) were also relatively common, whereas only one third of professionals conduct cognitive assessment. Scope of practice differed according to discipline, setting (oncology vs. outside oncology), and illness phase. The main barriers to providing psychological support were limited resources and funding, insufficient staff time or capacity, lengthy waitlists and costs, poor service coordination or integration, and other health professionals' lack of brain tumor‐specific experience or training.

A key finding was that approximately one third of professionals do not routinely screen for distress, which was most common for those working with PwBT across all illness phases. One interpretation of this finding is that distress levels of many PwBT are not monitored across the illness trajectory. The qualitative data highlighted that lack of time and resources were common in the hospital setting, with a focus on disease monitoring and medical care. Participants identified a lack of access to psychology and psychiatry services; hence, there may be concern about identifying distress without appropriate referral services. Distress levels can be particularly high for PwBT and caregivers at tumor recurrence,^31^ highlighting the need for routine screening and psychological intervention across the illness trajectory.

Importantly, rather than the findings reflecting a lack of adherence to guidelines for distress screening,^9^, ^10^, ^11^, ^12^, ^13^ some professionals within a multidisciplinary team may be more likely to conduct screening within their work scope than others. Screening may be conducted by referring professionals, or as part of a professional's own intake process. Psychologists and nurses were more likely to screen for distress than social workers. Further, 80% of other allied health and 63% of medical professionals “rarely” or “never” screen for distress (Supplementary Table 1), although cautious interpretation is needed due to low numbers of these professional groups. Distress screening was more common in the longer‐term management phase, consistent with tiered community models of psychosocial intervention whereby distress screening and needs assessment constitute Level 1 Universal Care.^32^

Some expected discipline differences emerged, with psychologists most likely to conduct cognitive assessments. These assessments were largely conducted by neuropsychologists outside oncology settings, in brain injury and neurosurgery units in which neuropsychological assessments are routine practice.^33^, ^34^ In line with ICCTF recommendations,^28^ Verbal Fluency, Trail Making Test, and memory tests were most frequently administered. Despite 33% of participants conducting cognitive assessments, only 14% provided cognitive rehabilitation, which is consistent with the qualitative data highlighting access to neuropsychology and rehabilitation as gaps in post‐treatment services.

Most professionals reported providing at least one session of psychological support to each PwBT, with the highest proportion providing 2‐8 sessions. These typically involved face‐to‐face psychoeducation and supportive counseling interventions, which may be flexibly employed by multiple professions. In the tiered model, these represent Level 2, Supportive Care Psychoeducation, indicated for individuals reporting mild distress levels,^32^ whereas those presenting with moderate‐to‐severe distress require Level 3–4 Extended or Specialist Counseling. Individuals presenting with very severe distress warrant Level 5 care, or referral to acute mental health services.^32^ Notably, several participants highlighted the challenges of linking PwBT to psychiatry services, particularly “*people with organic brain disease and concurrent mood disorders”* (P077). Overall, access to psychological support early post‐diagnosis/treatment was a major gap due to limited funding and resources.

Psychologists were less likely to provide psychological support for family members than nurses and social workers. This is potentially because access to psychology was often limited in acute treatment settings. Notably, professionals typically provided fewer support sessions to family members (1‐3 sessions) than PwBT (2‐8 sessions). Family support was more common in oncology settings, which may reflect the emphasis on carers within optimal care pathways for cancer and palliative care.^12^, ^35^ In research on family support, caregivers are typically involved in the same intervention as PwBT,^21^, ^22^, ^23^, ^36^ with few focused on the information and emotional support needs of family caregivers.^24^, ^36^ Accordingly, psychological support specific to family members was viewed as a gap by our respondents. Clinical guidelines for cancer highlight the need for caregiver support to extend from time of diagnosis through to bereavement.^9^

### 4.1 Clinical implications

The findings highlighted variations in psychological support according to discipline, setting, and illness phase. Professionals typically recognized the high and complex support needs of PwBT and family members, and how these differ from other cancer populations. Due to the combined cancer and neurological effects, PwBT may require varying and tailored combinations of medical care with rehabilitation, disability, mental health, and palliative care services. Yet, lack of care coordination or service integration and other professionals' lack of knowledge of brain tumor were barriers to receiving timely and appropriate psychological support. Professionals also perceived some PwBT find access to services difficult due to their tumor (benign vs. malignant), prognosis, or functional deficits. Individuals with benign tumor may not be eligible for some cancer support services, whereas those with malignant tumor may be unable to access rehabilitation and disability services due to their poor prognosis and expected functional decline. These findings reinforce the need for care coordination services (ideally conducted by neuro‐oncology specialists) to support PwBT and family members to navigate health and disability services across the care continuum.^37^ While care coordination is one way to ensure navigation and service needs are met, the varying scope of the different disciplines indicates the need for standardization and definition of roles within multidisciplinary teams at a service level. Clarity of roles at the service level can facilitate effective teamwork within care teams and improve self‐management of PwBT and family members.^38^, ^39^

### 4.2 Limitations

Although the survey was disseminated broadly across Australia via national multidisciplinary organizations, some states and disciplines (e.g., psychiatry & oncology) were underrepresented. This may be related to how our survey invitation defined supportive care. Nonetheless, participant numbers were highest from the three most populous Australian states (New South Wales, Vic, QLD) and disciplines were consistent with membership of Australia's psycho‐oncology network (>2000 members), largely comprised of psychologists, nurses and social workers.^40^ Due to the need for brevity, we did not assess timing of delivery for each psychological support practice or separately assess the frequency or nature of distress screening conducted with family members, or interventions specific to PwBT and family members. We also did not explore changes in service delivery due to the COVID‐19 pandemic.

Cross‐cultural comparisons of models of psychosocial care are recommended to obtain a wider perspective on psychosocial support practices of health professionals working with PwBT internationally and organizational and policy factors influencing implementation of psychosocial support services in practice. Research focused on gaining in‐depth understanding of the variations in access to psychosocial care according to tumor types, disease status, and socio‐cultural backgrounds is recommended, potentially through interviews with PwBT, family caregivers, and health professionals.

## 5 CONCLUSION

Despite some differences between disciplines and across settings and illness phase, most health professionals working with PwBT in this Australian study provide at least one session of psychological support, conduct distress screening, and support family members. Barriers to timely and quality psychosocial care identified for this population included limited resources and funding, insufficient staff time or capacity, lengthy waitlists and costs, poor service coordination, and lack of brain tumor‐specific experience or training. Further research on models of psychosocial care across settings would inform strategies to address organizational and policy factors impacting professionals' scope of practice.

## Supporting information

Supplementary Material 1

## Data Availability

The data that support the findings of this study are available from the corresponding author upon reasonable request.
