# Supplementary material for: Scoping the psychological support practices of Australian health professionals working with people with primary brain tumor and their families
Source: Psychooncology. 2022 Apr 2;31(8):1313–21. doi: 10.1002/pon.5929 (PMC9543201; doi:10.1002/pon.5929)
Supplement: Supplementary file 1 — Supplementary Material 1 [file PON-31-1313-s001.docx]

**SUPPLEMENTAL MATERIALS**

***Supplemental material 1 - survey (specific to questions regarding psychological support practices)***

Nature of Psychological Support and Supportive Care Provided to Adults with Brain Tumour

Q21: How often do you formally screen for psychological distress using a validated measure in adults with primary brain tumour? Please answer this in terms of the proportion of the clients you see with brain tumour.

- Never (0% of clients seen)
- Rarely (10-20% of clients seen)
- Sometimes (30-50% of clients seen)
- Most of the time (60-80% of clients seen)
- Always (~100% of clients seen)

Q22: What distress screening measure/s do you most typically use? (select all that apply)

- DASS-21
- DASS-42
- K-10
- HADS
- GAD-7
- Distress Thermometer
- Others (feel free to use widely known acronyms):_________________________________

Q23. Please indicate how or what method you typically use to conduct this screening (please indicate all that apply)

- Pencil and paper
- Oral questions via telephone
- Electronically during visits
- Electronically at home in advance of visits

Q24: How often do you conduct formal cognitive screening or assessment with adults with primary brain tumour? Please answer this in terms of the proportion of the clients you see with brain tumour.

- Never (0% of clients seen)
- Rarely (10-20% of clients seen)
- Sometimes (30-50% of clients seen)
- Most of the time (60-80% of clients seen)
- Always (~100% of clients seen)

Q25: What is the typical nature of cognitive assessment conducted with adults with primary brain tumour in your role?

- A single cognitive screening test (< 20 minutes)
- A brief cognitive battery assessing multiple domains (20-60 minutes)
- A full and comprehensive cognitive assessment (> 1 hour)

Q26: Please select or indicate the names of the cognitive tests or batteries you most typically use with adults with brain tumour (select all that apply):

- Repeatable Battery for the Assessment of Neuropsychological Status (RBANS)
- Wechsler Abbreviated Scale of Intelligence (WASI-II)
- Hopkins Verbal Learning Test-Revised (HVLT-R)
- Trail Making Test (TMT)
- Verbal Fluency Test / Controlled Oral Word Association
- Wechsler Adult Intelligence Scale (WAIS-IV)
- Wechsler Memory Scale (WMS-IV)
- Addenbrooke’s Cognitive Examination (ACE)
- Mini-Mental State Examination (MMSE)
- Montreal Cognitive Assessment (MoCA)
- Others (feel free to use widely known acronyms, e.g., TEA):_________________________

Q27: Separate to intake and assessment sessions, how often do you provide adults (18+ years) with primary brain tumour with at least one session of psychological support or therapy focused on supporting their adjustment to the emotional, cognitive, and/or behavioural effects of brain tumour? Please answer this in terms of the proportion of the clients you see with brain tumour.

- Never (0% of clients seen)
- Rarely (10-20% of clients seen)
- Sometimes (30-50% of clients seen)
- Most of the time (60-80% of clients seen)
- Always (~100% of clients seen)

Q28: What is the most common number of psychological support sessions you provide to adults with brain tumour?

- A single session (< 30 minutes)
- A single session (> 30 minutes)
- 2-3 sessions
- 4-5 sessions
- 6-8 sessions
- 9-10 sessions
- 10-20 sessions
- 20 sessions
- Session number varies. Please describe: ____________________

Q29: How often do you provide psychological support to family members of adults with primary brain tumour? Please answer as a proportion of the clients you see with brain tumour.

- Never (0% of clients)
- Rarely (10-20% of clients)
- Sometimes (30-50% of clients)
- Most of the time (60-80% of clients)
- Always (~100% of clients)

Q30: What is the most common number of psychological support sessions you provide to family members?

- A single session (< 30 minutes)
- A single session (> 30 minutes)
- 2-3 sessions
- 4-5 sessions
- 6-8 sessions
- 9-10 sessions
- 10-20 sessions
- >20 sessions
- Session number varies. Please describe: ____________________

Q31: Through which mode of delivery do you most typically provide psychological support to people with brain tumour and/or family members?

- Telephone
- Videoconferencing
- Face-to-face
- A mix of telehealth and face-to-face
- Other: ____________________

Q32: Please indicate the types of support or interventions you most typically provide to people with brain tumour and/or family members (tick all that apply)

- Psycho-education or information about brain tumour and the cognitive, emotional, and behavioural effects
- Cognitive rehabilitation (i.e., cognitive remediation and/or compensatory strategies for managing memory and other cognitive problems). If yes, ask Q34
- Supportive counselling (i.e., counselling to assist with adjustment to diagnosis, grief and the consequences of brain tumour)
- Behaviour management (e.g., positive behavioural support to individuals, families, and/or formal carers)
- Psychological therapies (e.g., cognitive behavioural therapy to treat depression and anxiety in the context of brain tumour)
- Family-based therapy or relationship counselling
- Group therapy involving other people with brain tumour, cancer, or brain injury
- Other: _______________________

Q33: Which kinds of psychological therapies do you most typically use? (select all that apply)

- Cognitive Behavioural Therapy
- Acceptance and Commitment Therapy
- Motivational Interviewing
- Mindfulness-Based Cognitive Behaviour Therapy
- Solution-Focused Therapy
- Narrative Therapy
- Dignity Therapy
- Other: __________________________________

Q34: Which format and kinds of cognitive rehabilitation interventions do you most typically use? (select all that apply)

- Individual cognitive remediation (e.g., attention process training, computerised exercises)
- Individual compensatory training (e.g., teaching people how to use memory strategies)
- Group-based cognitive rehabilitation
- A mix of the above
- Other: _________________

***Supplementary Table 1. Frequency of screening for psychological distress across disciplines (n = 107)***

|  | **Frequency of screening for psychological distress*** n(%)** | | | | |
| --- | --- | --- | --- | --- | --- |
|  | **Infrequently** | | | **Frequently** | |
|  | Never  (0%) | Rarely  (10-20%) | Sometimes  (30-50%) | Almost always  (60-80%) | Always  (~100%) |
| **Discipline** | | | | | |
| Psychologists (n = 45)  Clinical/health (n=24)  General (n=3)  Neuro (n=18) | 3(6.7%)  1(4.2%)  1(33.3%)  2(11.1%) | 4(8.9%)  3(12.5%)  0(0%)  0(0%) | 6(13.3%)  5(20.9%)  0(0%)  1(5.6%) | 11(24.4%)  8(33.3%)  0(0%)  3(16.7%) | 21(46.7%)  7(29.2%)  2(66.7%)  12(66.7%) |
| Nurses (n = 21) | 3(14.3%) | 0(0%) | 2(9.5%) | 8(38.1%) | 8(38.1%) |
| Social workers (n = 11) | 6(54.5%) | 1(9.1%) | 1(9.1%) | 3(27.3%) | 0(0%) |
| Other allied health* (n=10) | 5(50%) | 3(30%) | 1(10%) | 0(0%) | 1(10%) |
| Medicine (n = 14)  Oncologists (n = 9)  Psychiatry (n = 3)  GP (n=1)  Surgeon (n=1) | 6(42.9%)  3(33.3%)  1(33.3%)  1(100%)  1(100%) | 3(21.4%)  1(11.1%)  2(66.7%)  0(0%)  0(0%) | 2(14.3%)  2(22.2%)  0(0%)  0(0%)  0(0%) | 3(21.4%)  3(33.3%)  0(0%)  0(0%)  0(0%) | 0(0%)  0(0%)  0(0%)  0(0%)  0(0%) |
| Other (n=6)** | 3(50%) | 0 (0%) | 1(16.7%) | 1(16.7%) | 1(16.7%) |
| **Clinical population/setting** | | | | | |
| Oncology (n=87) | 21(24.1%) | 11(12.6%) | 12(13.8%) | 20(23%) | 23(26.4%) |
| Not oncology (n=20) | 5(25%) | 0(0%) | 1(5%) | 6(30%) | 8(40%) |
| **Phase of illness** | | | | | |
| Early post-diagnosis/acute treatment (n=23) | 6(26.1%) | 1(4.3%) | 3(13%) | 8(34.8%) | 5(21.7%) |
| Long-term post-treatment and end of life (n=28) | 4(14.3%) | 2(7.1%) | 2(7.1%) | 5(17.9%) | 15(53.6%) |
| Across all phases (n=56) | 16(28.6%) | 8(14.3%) | 8(14.3%) | 13(23.2%) | 11(19.6%) |

*8 Occupational therapists, 1 speech pathologist, 1 exercise physiologist; **1 palliative care specialist, 2 counsellors, 2 representatives of cancer support organisations; 1 peer supporter. For the statistical analysis, the answers ‘never’, ‘rarely’ and ‘sometimes’ were classified as infrequently while ’almost always’ and ‘always’ as frequently.; ***Frequency of psychological support practices was rated as a proportion of all clients seen with brain tumour, as follows: never - 0% of clients seen; rarely - 10-20% of clients seen; sometimes – 30-50% of clients seen; almost always – 60-80% of clients seen; always - ~100% of clients seen.

***Supplementary Table 2. The most commonly used measures to screen for distress***

| **Distress Measures** | **Responses** | |
| --- | --- | --- |
|  | **N** | **%** |
| Distress Thermometer (DT) | 44 | 41.1% |
| Depression Anxiety Stress Scale 21 (DASS-21) | 27 | 25.2% |
| Hospital Anxiety and Depression Scale (HADS) | 15 | 14% |
| Generalised Anxiety Disorder Assessment (GAD-7) | 9 | 8.4% |
| Depression Anxiety Stress Scale 42 (DASS-42) | 5 | 4.7% |
| Kessler Psychological Distress Scale (K-10) | 4 | 3.7% |
| Clinical interview | 3 | 2.8% |
| Patient Health Questionnaire 9 (PHQ-9) | 3 | 2.8% |
| Beck Depression Inventory (BDI-II) | 3 | 2.8% |
| Impact of Event Scale – Revised (IES-R) | 2 | 1.9% |
| Beck Anxiety Inventory (BAI-II) | 2 | 1.9% |
| Patient Health Questionnaire 4 (PHQ-4) | 1 | 0.9% |
| Aged care assessment | 1 | 0.9% |
| Self-Rating Anxiety Scale (SAS and eSAS) | 1 | 0.9% |
| The Minnesota Multiphasic Personality Inventory (MMPI) | 1 | 0.9% |
| Geriatric Depression Scale 15 (GDS 15) | 1 | 0.9% |
| State-Trait Anxiety Inventory (STAI) | 1 | 0.9% |

*participants could indicate more than one answer;

***Supplementary Table 3. Frequency of cognitive screening and/or assessment (n = 107)***

|  | **Frequency of cognitive screening and/or assessment n(%)*** | | | | |
| --- | --- | --- | --- | --- | --- |
|  | **Infrequently** | | | **Frequently** | |
|  | Never  (0%) | Rarely  (10-20%) | Sometimes  (30-50%) | Almost always  (60-80%) | Always  (~100%) |
| **Discipline** | | | | | |
| Psychologists (n = 45)  Clinical/health (n=24)  General (n=3)  Neuro (n=18) | 15(33.3%)  11(45.8%)  2(66.7%)  2(11.1%) | 11 (24.4)  9(37.5%)  1(33.3%)  1(5.5%) | 5 (11.1%)  4(16.7%)  0(0%)  1(5.5%) | 7 (15.6%)  0(0%)  0(0%)  7(38.9%) | 7(15.6%)  0(0%)  0(0%)  7(38.9%) |
| Nurses (n = 21) | 12(57.1%) | 5(23.8%) | 2(9.5%) | 2(9.5%) | 0(0%) |
| Social workers (n = 11) | 8(72.7%) | 1(9.1%) | 2(18.2%) | 0(0%) | 0(0%) |
| Other allied health (n=10) | 0(0%) | 5(50%) | 2(20%) | 3(30%) | 0(0%) |
| Medicine (n = 14)  Oncologists (n = 9)  Psychiatry (n = 3)  GP (n=1)  Surgeon (n=1) | 4(28.6%)  3(33.3%)  0(0%)  1(100%)  1(100%) | 5(35.7%)  4(44.4%)  1(33.3%)  0(0%)  0(0%) | 3(21.4%)  1(11.1%)  1(33.3%)  0(0%)  0(0%) | 2(14.3%)  1(11.1%)  1(33.3%)  0(0%)  0(0%) | 0(0%)  0(0%)  0(0%)  0(0%)  0(0%) |
| Other (n=6) | 4(66.7%) | 2(33.3%) | 0(0%) | 0(0%) | 0(0%) |
| **Clinical population/setting** | | | | | |
| Oncology (n=87) | 37(42.5%) | 25(28.7%) | 13(14.9%) | 9(10.3%) | 3(3.4%) |
| Not oncology (n=20) | 6(30%) | 4(20%) | 1(5%) | 5(25%) | 4(20%) |
| **Phase of illness** | | | | | |
| Early post-diagnosis/acute treatment (n=23) | 10(43.5%) | 4(17.4%) | 2(8.7%) | 6(26.1%) | 1(4.3%) |
| Long-term post-treatment and end of life (n=28) | 7(25%) | 7(25%) | 4(14.3%) | 4(14.3%) | 6(21.4%) |
| Across all phases (n=56) | 26(46.4%) | 18(32.1%) | 8(14.3%) | 4(7.1%) | 0(0%) |

*Frequency of psychological support practices was explained as follow: never - 0% of clients seen; rarely - 10-20% of clients seen; sometimes – 30-50% of clients seen; almost always – 60-80% of clients seen; always - ~100% of clients seen.

***Supplementary Table 4. The most commonly used cognitive assessment measures***

| **Cognitive Assessment Measures** | **Responses** | |
| --- | --- | --- |
|  | **N** | **%** |
| Verbal Fluency Test / Controlled Oral Word Association | 19 | 17.8% |
| Trail Making Test (TMT) | 17 | 15.9% |
| Wechsler Adult Intelligence Scale (WAIS-IV) | 14 | 13.1% |
| Montreal Cognitive Assessment (MoCA) | 14 | 13.1% |
| Wechsler Memory Scale (or its subscales) (WMS-IV) | 14 | 13.1% |
| Mini-Mental State Examination (MMSE) | 12 | 11.2% |
| Rey Complex Figure Test (RCFT) | 8 | 7.5% |
| Hopkins Verbal Learning Test-Revised (HVLT-R) | 4 | 3.7% |
| California Verbal Learning Test (CVLT) | 4 | 3.7% |
| Boston Naming Test (BNT) | 4 | 3.7% |
| The Delis–Kaplan Executive Function System (D-KEFS) | 4 | 3.7% |
| Rey Auditory Verbal Learning Test RAVLT- 4 | 4 | 3.7% |
| Repeatable Battery for the Assessment of Neuropsychological Status (RBANS) | 3 | 2.8% |
| Addenbrooke’s Cognitive Examination (ACE) | 3 | 2.8% |
| Color-Word Interference Test (CWIT) or The Stroop Color and Word Test (SCWT) | 3 | 0.9% |
| Wisconsin Card Sorting Test (WCST) | 2 | 1.9% |
| Hayling sentence completion | 2 | 1.9% |
| Barry Rehabilitation Inpatient Screening of Cognition (BRISC) | 2 | 1.9% |
| Test of Premorbid Functioning (TOPF) | 2 | 1.9% |
| Wechsler Abbreviated Scale of Intelligence (WASI-II) | 1 | 0.9% |
| Tea and toast | 1 | 0.9% |
| Cognitive estimates | 1 | 0.9% |
| Frontal assessment battery | 1 | 0.9% |
| Austin Maze Test | 1 | 0.9% |
| Judgment of Line Orientation (JLO) | 1 | 0.9% |
| Modified Token Test | 1 | 0.9% |
| Benton Facial Recognition | 1 | 0.9% |
| The Wechsler Test of Adult Reading (WTAR) | 1 | 0.9% |
| Rowland Universal Dementia Assessment Scale (RUDAS) | 1 | 0.9% |
| The General Practitioner assessment of Cognition (GPCOG) | 1 | 0.9% |
| Tower of London (TOL) | 1 | 0.9% |
| Selected Western Aphasia Battery subtests and other "clinical" language tests as required | 1 | 0.9% |

***Supplementary Table 5. Frequency of providing psychological support (> 1 session) to people with brain tumor (n = 103)***

|  | **Frequency of providing psychological support session(s) n(%)*** | | | | |
| --- | --- | --- | --- | --- | --- |
|  | **Infrequently** | | | **Frequently** | |
|  | Never  (0%) | Rarely  (10-20%) | Sometimes  (30-50%) | Almost always  (60-80%) | Always  (~100%) |
| **Discipline** | | | | | |
| Psychologists (n = 45)  Clinical/ health (n=24)  General (n=3)  Neuro (n=18) | 1 (2.2%)  0(0%)  0(0%)  1(5.5%) | 2(4.4%)  2(8.3%)  0(0%)  0(0%) | 7(15.6%)  2(8.3%)  0(0%)  5(27.8%) | 19(42.2%)  10(41.7%)  1(33.3%)  8(44.4%) | 16(35.6%)  10(41.7%)  2(66.7%)  4(22.2%) |
| Nurses (n = 19) | 3(15.8%) | 3(15.8%) | 5(26.3%) | 6(31.6%) | 2(10.5%) |
| Social workers (n = 9) | 0(0%) | 0(0%) | 2(18.2%) | 6(54.5%) | 2(22.2%) |
| Other allied health (n=9) | 0(0%) | 0(0%) | 2(22.2%) | 5(55.6%) | 2(22.2%) |
| Medicine (n = 13)  Oncologists (n = 8)  Psychiatry (n = 3)  GP (1)  Surgeon (1) | 3(23.1%)  1(12.5%)  0(0%)  1(100%)  1(100%) | 3(23.1%)  3(37.5%)  0(0%)  0(0%)  0(0%) | 2(15.4%)  2(25%)  0(0%)  0(0%)  0(0%) | 2(15.4%)  1(12.5%)  1(33.3%)  0(0%)  0(0%) | 3(23.1%)  1(12.5%)  2(66.7%)  0(0%)  0(0%) |
| Other (n=6) | 1 (16.7%) | 0(0%) | 1(16.7%) | 2(33.3%) | 2(33.3%) |
| **Clinical population** | | | | | |
| Oncology (n = 84) | 7(8.3%) | 7(8.3%) | 15(17.9%) | 31(36.9%) | 24(28.6%) |
| Not oncology (n=19) | 1(5.3%) | 1(5.3%) | 4(21.1%) | 9(47.4%) | 4(21.1%) |
| **Phase of illness** | | | | | |
| Early post-diagnosis/acute treatment (n=23) | 4(17.4%) | 2(8.7%) | 7(30.4%) | 8(34.8%) | 2(8.7%) |
| Long-term post-treatment and end of life (n=27) | 0(0%) | 0(0%) | 6(22.2%) | 12(44.4%) | 9(33.3%) |
| Across all phases (n=53) | 4(7.5%) | 6(11.3%) | 6(11.3%) | 20(37.7%) | 17(32.1%) |

*Frequency of psychological support practices was explained as follow: never - 0% of clients seen; rarely - 10-20% of clients seen; sometimes – 30-50% of clients seen; almost always – 60-80% of clients seen; always - ~100% of clients seen.

***Supplementary Table 6. Frequency of providing psychological support (> 1 session) to family members (n = 103)***

|  | **Frequency of proving psychological support to family members n(%)*** | | | | | |
| --- | --- | --- | --- | --- | --- | --- |
|  | **Infrequently** | | | **Frequently** | | |
|  | Never (0%) | Rarely  (10-20%) | Sometimes (30-50%) | Almost always  (60-90%) | Always (~100%) | |
| **Discipline** | | | | | |  |
| Psychologists (n = 45)  Clinical/ health (n=24)  General (n=3)  Neuro (n=18) | 11 (24.4%)  6(25%)  0(0%)  5(27.8%) | 9(20%)  5(20%)  2(66.7%)  2(11.1%) | 13(28.9%)  6(25%)  1(33.3%)  6(33.3%) | 11(24.4%)  7(30%)  0(0%)  4(22.2%) | 1(2.2%)  0(0%)  0(0%)  1(5.56%) | |
| Nurses (n = 19) | 0(0%) | 2(10.5%) | 4(21.1%) | 12(63.2%) | 1(5.3%) | |
| Social workers (n = 11) | 0(0%) | 1(9.1%) | 2(18.2%) | 7(63.6%) | 1(9.1%) | |
| Other allied health (n=9) | 0(0%) | 1(11.1%) | 3(33.3%) | 5(55.6%) | 0(0%) | |
| Medicine (n = 13)  Oncologists (n = 8)  Psychiatry (n = 3)  GP (1)  Surgeon (1) | 1(7.7%)  1(12.5%)  0(0%)  0(0%)  0(0%) | 4(30.8%)  3(37.5%)  0(0%)  0(0%)  1(100%) | 5(38.5%)  2(25%)  2(66.7%)  1(100%)  0(0%) | 3(23.1%)  2(25%)  1(33.3%)  0(0%)  0(0%) | 0(0%)  0(0%)  0(0%)  0(0%)  0(0%) | |
| Other | 0(0%) | 1(16.7%) | 3(50%) | 0(0%) | 2(33.3%) | |
| **Clinical population/setting** | | | | | |  |
| Oncology (n=84) | 6(7.1%) | 15(17.9%) | 23(27.4%) | 35(14.7%) | 5(6%) | |
| Not oncology (n=19) | 6(31.6%) | 3(15.8%) | 7(36.8%) | 3(15.8%) | 0(0%) | |
| **Phase of illness** | | | | | |  |
| Early post-diagnosis/acute treatment (n=23) | 5(21.7%) | 4(17.4%) | 7(30.4%) | 6(26.1%) | 1(4.3%) | |
| Long-term post-treatment and end of life (n=27) | 4(14.8%) | 4(14.8%) | 10(37%) | 8(29.6%) | 1(3.7%) | |
| Across all phases (n=53) | 3(5.7%) | 10(18.9%) | 13(24.5%) | 24(45.3%) | 3(5.7%) | |

*Frequency of psychological support practices was explained as follow: never - 0% of clients seen; rarely - 10-20% of clients seen; sometimes – 30-50% of clients seen; almost always – 60-80% of clients seen; always - ~100% of clients seen.

***Supplementary Table 7. Most typical mode of delivery of psychological support to adults with brain tumour and/or family members***

| **Mode of delivery** | **N** | | **%** | |
| --- | --- | --- | --- | --- |
| Telephone | 5 | 4.7% | |  |
| Videoconferencing | 1 | 0.9% | |  |
| Face-to-face | 51 | 47.7% | |  |
| A mix of telehealth and face-to-face | 30 | 28% | |  |
| Missing | 20 | 18.7% | |  |

***Supplementary Table 8. Types of psychological support or interventions typically provided***

| **Types of support/interventions** | **N** | **%** |
| --- | --- | --- |
| Psycho-education or information about brain tumour and the cognitive, emotional, and behavioural effects | 79 | 24.9% |
| Cognitive rehabilitation (i.e., cognitive remediation and/or compensatory strategies for managing memory and other cognitive problems) | 45 | 14.2% |
| Supportive counselling (i.e., counselling to assist with adjustment to diagnosis, grief and the consequences of brain tumour) | 74 | 23.3% |
| Behaviour management (e.g., positive behavioural support to individuals, families, and/or formal carers) | 45 | 14.2% |
| Psychological therapies (e.g., cognitive behavioural therapy to treat depression and anxiety in the context of brain tumour) | 35 | 11.0% |
| Family-based therapy or relationship counselling | 18 | 5.7% |
| Group therapy involving other people with brain tumour, cancer, or brain injury | 12 | 3.8% |
| Other | 9 | 2.8% |

*Multiple responses were permitted

***Supplementary Table 9. Types of psychological therapy interventions typically provided***

| **Types of psychological interventions** | **N** | **%** |
| --- | --- | --- |
| Cognitive Behavioural Therapy | 37 | 18.9% |
| Acceptance and Commitment Therapy | 43 | 21.9% |
| Motivational Interviewing | 29 | 14.8% |
| Mindfulness-Based Cognitive Behaviour Therapy | 23 | 11.7% |
| Solution-Focused Therapy | 22 | 11.2% |
| Narrative Therapy | 16 | 8.2% |
| Dignity Therapy | 10 | 5.1% |
| Other | 16 | 8.2% |

*Multiple responses were permitted

***Supplementary Table 10. Types of cognitive rehabilitation typically provided***

| **Types of cognitive rehabilitation provided** | **N** | **Percent** |
| --- | --- | --- |
| Individual cognitive remediation (e.g., attention process training, computerised exercises) | 7 | 13.0% |
| Individual compensatory training (e.g., teaching people how to use memory strategies) | 39 | 72.2% |
| Group-based cognitive rehabilitation | 4 | 7.4% |
| Other | 4 | 7.4% |

*Multiple responses were permitted

**Supplementary Table 11. Coding of open-ended question regarding perceived barriers and gaps in psychological support (n = 76)**

| Categories and subcategories | N(%) | Example quotes |
| --- | --- | --- |
| System-level barriers/gaps  Limited resources and funding for services specific to people with brain tumour and families. | 21 (28) | - Limited support for carers who bear the biggest burden. Little cognitive support for primary CNS patients - Hospitals have minimal on-site psychological resources for people with brain tumour. - Lack of suitable accommodation and respite options for patients with complex behavioral change - Severely under resourced to refer internally for psychological support either psychologist or psycho-oncology in outpatient setting. - Lack of brain cancer specialist nurse (like for other types of cancers). The psychological and psychosocial needs of brain cancer patients may be missed. - Lack of supports available to adults with brain tumour, particularly those under the age of 65. - More resources, and intervention for CALD populations |
| Insufficient time or capacity within staffing | 20 (26) | - Not enough psychology EFT, not all brain tumour patients get seen or get enough support. - Brief/time limited service so assessment, family work and long-term therapy are out of scope - Lack of time and opportunity is a barrier in the hospital and medical settings - people's energies are taken up with appointments. - Lack of staff, high case load and complex cases, nil neuropsychologists employed |
| Post-treatment rehabilitation and support services | 19 (25) | - Neuropsychological testing services post-treatment - Post treatment re: return to work and driving as this is usually a sign of independence and identity for and there is limited guidance for patients and their family - Care co-ordinators in each healthcare setting to support patients & carers during & long-term post-treatment. - Allied health support (social work, physio, OT) in outpatient clinic - Access to neuropsychology, neuropsychiatry and psychology |
| Lengthy waitlists and costs associated with psychological assessments and interventions | 18 (24) | - Private psychology is expensive and I rarely receive referrals for people early in the journey. - Limited resources for psycho-social care, meaning long waiting times for appointments. - Not enough neuro-psychology services within public health, private NeuroPsych too expensive - The gap cost involved with community psychology is prohibitive in some cases, even with a mental health care plan. - Beyond our service, we hear that it can be difficult to see psychology in the treatment centres, with long waiting times, and beyond that, costly in the community. |
| Poor coordination or integration between services | 17 (22) | - If they don't come through the brain injury clinic they fall through the gaps and never get access to anyone - Lack of buy in from inpatient and community mental health services to assist in managing high risk patients with mania, psychosis, suicidality - Disability services are not responsive to progressive and deteriorating situation and access to palliative care varies across different regions. - Not all clients with brain tumour are referred to our services from the medical model - it is a random referral process from a nurse or a specialist or community member - People with brain tumour typically do not receive referral or support to access disability supports from the health sector. |
| Lack of staff with training or experience with brain tumour | 16 (21) | - Lack of clinical psychologists trained to provide therapy adapted for people with cognitive impairment. - Psychologists/psychiatrist lack of understanding of the nature and impacts of brain tumour. - Cancer focused psychologists aren't always equipped to understand the cognitive effects of brain tumour. - Limited experience and knowledge in working with people with brain tumours |
| Lack of awareness of services available or how to access these | 10 (13) | - Not knowing what's available - Unclear referral processes and knowledge of support services available. - Knowing pathways |
| Greater support for family members | 8 (11) | - Support for their partners and family members - Supportive psychotherapy and education to children of adults with brain tumours - Families would also benefit from more access to psychology - Carer support programs which are specific to primary brain tumours rather than dementia/brain injury. |
| Barriers related to people with brain tumor |  |  |
| Variations in access due to tumor characteristics and prognosis | 10 (13) | - ABI services (who might be best placed to provide psychological support or refer to skilled professionals) often don't accept people with brain tumour. - Applying for NDIS funding in a time effective manner. This is often a lengthy process in a client group with often short prognosis. - NDIS applications often take too long and are not responsive to progressive and deteriorating situation. - Limited supports for patients within the GBM population. Often leave hospital with minimal impairments and don't fit into criteria for follow up but then decline rapidly in the community |
| Acceptance of counselling and functional deficits | 9 (12) | - Many services must be self-initiated by the patient, which can be a barrier for their ongoing attendance and participation - How cognitive impairments impact psychological interventions is often not considered. - Acceptance, readiness to grieve, cognitive/behavioural issues, reduced insight - Cognitive ability of the person can mean communication is difficult - Significant cognitive and/or communication impairment limits the interventions that can be used. |
| Travel and transport issues | 4 (5) | - Lack of access due to patients unable to travel - Limited number of support services in the state. Difficult to access for out-of-area patients - needs for travel/transportation - Limited specialised psychological support for end-of-life care when patients are no longer able to travel to health care centres. |
|  |  |  |
